# Recent progress in women’s mental health research and future priorities

**DOI:** 10.1192/j.eurpsy.2025.146

**Published:** 2025-08-26

**Authors:** F. Thibaut

**Affiliations:** 1 Psychiatry and Addictive Disorders, University Paris Cité; 2 Psychiatry and Addictive Disorders, University Hospital Cochin; 3 INSERM U1266, Institute of Psychiatry and Neurosciences, Paris, France

## Abstract

**Introduction:**

Women face a greater incidence of mental health issues than men, stemming from societal expectations, gender stereotypes, organizational structures that prioritize male leadership, and the need to balance work responsibilities with home life. Research indicates that women are twice as likely to suffer from depression, generalized anxiety disorder, and post-traumatic stress disorder (PTSD) compared to men. They are also more likely to battle eating disorders.

**Methods:**

The authors will review the current literature on this topic.

**Results:**

43% of female executives experience burnout, compared to 31% of their male counterparts (Mc Kinsey 2024). Research is definitely needed to better understand the pathophysiology and socioeconomic mechanisms that drive sex-specific risk factors of psychological disorders in women. There is also an urgent need for studies that addressed the specific needs of women including insights around: the biological, life stage, socioeconomic, political and cultural factors associated with being female that have a significant impact on women’s mental health.

**Conclusion:**

A mental health reform is necessary to prevent mental illnesses in women, provide specific care to vulnerable women and increase the overall well-being of women living with existing mental health conditions.

**Disclosure of Interest:**

None Declared

